# Immediate Effects of Delayed Auditory Feedback on Stuttering: A Systematic Review and Meta‐Analysis of Literature Published 2000–2024

**DOI:** 10.1111/1460-6984.70283

**Published:** 2026-06-24

**Authors:** Daichi Iimura, Takuma Yamamoto, Osamu Ishida

**Affiliations:** ^1^ Institute of Human Sciences University of Tsukuba Tsukuba Ibaraki Japan; ^2^ Graduate School of Intelligent and Mechanical Interaction Systems University of Tsukuba Tsukuba Ibaraki Japan; ^3^ Faculty of Education Chiba University Chiba Japan

**Keywords:** delayed auditory feedback, meta‐analysis, stuttering, disfluency, systematic review

## Abstract

**Purpose:**

This systematic review and meta‐analysis evaluated the fluency‐ enhancing effect of DAF alone in individuals with developmental stuttering.

**Methods:**

Following PRISMA 2020 guidelines, we searched multiple databases for studies published between 2000 and 2024. Eligible studies examined DAF conditions applied to speech tasks with stuttering‐related outcomes. Meta‐analyses were conducted using a random‐effects model, with subgroup analyses by disfluency type, delay time, speech task, stuttering severity, and participant age.

**Results:**

Of the 194 records screened, eight studies involving a total of 98 participants in total met the inclusion criteria, and five studies involving 61 participants were eligible for quantitative synthesis. Each study included 8–20 participants ranging from school‐age children to adults. Most participants were male, and stuttering severity ranged from mild to severe. DAF conditions were evaluated using oral reading and spontaneous speech/monologue tasks. Meta‐analysis revealed no significant overall benefit of DAF compared with normal auditory feedback (NAF; mean difference = –1.46, 95% CI [–4.83, 1.91]).

**Conclusion:**

DAF alone does not consistently reduce disfluencies; however, specific populations and conditions may derive greater benefits from it. Larger, well‐controlled studies are needed to clarify its therapeutic potential and clinical applications.

**WHAT THIS PAPER ADDS:**

*What is already known on this subject*

- Delayed auditory feedback (DAF) has been reported to improve fluency in people who stutter and is used in several assistive devices. However, its independent effect remains unclear because DAF is often combined with other altered auditory feedback conditions.

*What this study adds to existing knowledge*

- This systematic review and meta‐analysis evaluated the exclusive effect of DAF on stuttering. The results indicate that DAF alone does not consistently reduce disfluency compared with NAF, although certain conditions (e.g., shorter delays or reading tasks) may show greater benefits.

*What are the potential or actual clinical implications of this work?*

- Clinicians should interpret the fluency‐enhancing effects of DAF cautiously when used alone. Further well‐controlled studies are needed to determine which individuals and speech contexts may benefit most from DAF‐based interventions.

## 1 Introduction

Developmental stuttering is a speech fluency disorder characterized by repetitions, prolongations, and blocks, typically occurring at the beginning of utterances (Guitar 2025). A defining feature of stuttering is its variability across time and situations (Constantino et al. 2016; Ortiz‐Alvarez and Arenas 2025; Tichenor and Yaruss 2021). One well‐documented factor influencing disfluency is altered auditory feedback with a delay, known as delayed auditory feedback (DAF; Andrade and Juste 2011; Howell 2004; Lee 1950; Naylor 1953; Lincoln et al. 2006). DAF has also been examined in intervention studies (Baxter et al. 2015; Johnson et al. 2023) using devices such as SpeechEasy, Pocket Speech Lab, SmallTalk, Fluency Enhancer, Digital Speech Aid, Edinburgh Masker, and in‐ear fluency devices (e.g., Armson and Kiefte 2008; Baxter et al. 2015; Foundas et al. 2013; Pollard et al. 2009). Many of these devices combine DAF with frequency‐altered feedback (FAF).

The mechanisms underlying disfluency reduction with DAF remain debated (Chon et al. 2021; Foundas et al. 2013; Howell 2004; Kalinowski and Saltuklaroglu 2003; Lincoln et al. 2006). A widely accepted explanation is that DAF induces a reduction in speech rate or prolonged speech (Daliri et al. 2025), particularly at longer delays (Ryan and Ryan 1995; Ryan and Van Kirk 1974). According to the EXPLAN theory, even short delays may slow down speech by preventing overly rapid production when cognitive–linguistic planning lags behind motor execution (Howell 2004; Howell and Au‐Yeung 2002). Altered auditory feedback may therefore provide additional time for speech planning to prevent or correct errors. At shorter delays, DAF may also function as a secondary speech signal, similar to choral speech (Kalinowski and Dayalu 2002; Saltuklaroglu et al. 2009). This mechanism is thought to support the integration of speech production and perception through the mirror neuron system, although less effectively than true choral speech (Saltuklaroglu et al. 2009). Other studies have examined kinematic or acoustic variability under DAF compared with NAF (Chon et al. 2021; Daliri et al. 2025). Although many theories emphasize reduced speech rate as the primary factor, fluency enhancement is also observed during fast speech under short‐delay DAF (50–75 ms), suggesting that slower speech is not always necessary (Kalinowski et al. 1993; Kalinowski and Stuart 1996). These findings indicate that the fluency‐enhancing effects of DAF are multidimensional and may relate to subtypes of stuttering, for example, those associated with central auditory processing (Foundas et al. 2013; Picoloto et al. 2017).

Despite reports suggesting that DAF benefits individuals who stutter, its effectiveness remains debated (Andrade and Juste 2011; Fiorin et al. 2021; Foundas et al. 2013). Some studies have failed to replicate fluency‐enhancing effects (Alqhazo and Alkhamaiseh 2025; Chon et al. 2021). Variability in DAF outcomes has been linked to factors such as stuttering severity (Fiorin et al. 2021; Foundas et al. 2013; Sparks et al. 2002; Unger et al. 2012), delay time (Goldiamond 1965; Kalinowski and Stuart 1996; Lincoln et al. 2006), and speech task type (Armson et al. 2006; Armson and Stuart 1998; Foundas et al. 2013; Lincoln et al. 2006). However, the combined influence of these factors has not been systematically examined. A further complication is that DAF is often implemented together with FAF (Andrade and Juste 2011; Lincoln et al. 2006), making it difficult to isolate the effect of DAF alone. Consequently, previous findings have been inconsistent. To address this gap, the present systematic review and meta‐analysis evaluated the effect of DAF alone on reducing stuttering disfluency. The review was restricted to studies published from 2000 onward because, at the time the review was designed, we expected to identify a sufficient number of studies for synthesis. Furthermore, earlier studies may have demonstrated greater methodological variability (Andrade and Juste 2011).

## 2 Methods

### 2.1 Literature Search Strategy

Following the Preferred Reporting Items for Systematic Reviews and Meta‐Analyses (PRISMA) 2020 guidelines (Page et al. 2021), a multi‐database search was conducted in Web of Science, PubMed, PsycINFO, and Education Resources Information Center (ERIC). The search, completed on 19 October 2024, targeted studies published between 2000 and 2024 using the terms ‘stutter’ OR ‘stamm*’ AND ‘delay* ‘auditory feedback’ OR ‘DAF.’ A manual search of the reference lists of previous reviews (Andrade and Juste 2011; Lincoln et al. 2006) and additional sources (i.e., Google Scholar) was also conducted to identify potentially eligible articles. Search results were exported to EndNote X9.3.3 (Windows), and duplicates were manually removed.

### 2.2 Inclusion and Exclusion Criteria

Studies were eligible if they (a) included individuals with developmental stuttering, (b) applied a speech task under DAF, and (c) measured speech disfluencies (stuttering‐like disfluencies [SLD], other disfluencies [OD], or total disfluencies). Exclusion criteria were as follows: (a) conference papers, reviews, commentaries, or dissertations; (b) non‐developmental stuttering samples; (c) absence of DAF speech tasks; (d) no disfluency outcomes reported; (e) combined fluency‐enhancing conditions (e.g., DAF with FAF or masking); (f) sample size < 5; and (g) non‐English articles.

### 2.3 Study Selection

Article selection followed a two‐stage PRISMA 2020 screening process. In Stage 1, titles and abstracts were screened against the eligibility criteria. Articles of uncertain relevance proceeded to Stage 2, where full texts were reviewed. Two authors (OI, TY) independently evaluated each article, and Cohen's kappa (κ) was calculated to assess inter‐rater reliability. Disagreements were resolved through discussion.

### 2.4 Data Extraction

Data were extracted for the following dimensions: study information (author, year of publication), participants (sample size, age, gender, inclusion criteria, stuttering severity), procedures (speech task, delay time, control condition, independent variables), and outcomes (main findings, and statistical values such as means and standard deviations [SD] of disfluencies). One author (DI) charted data from the included studies, and all authors subsequently reviewed the extracted data. Any discrepancies were resolved through discussion.

### 2.5 Quality Assessment

Study quality was assessed using the index developed by Andrade and Juste (2011). One point was awarded for each of the following: the use of blinding or inter‐rater agreement, inclusion of a control group, quantitative data analysis, statistical testing, and a longitudinal design. Scores ranged from 0 (lowest quality) to 5 (highest). Two authors (OI, TY) independently rated the studies, resolving disagreements with a third author (DI).

### 2.6 Data Analysis and Statistical Analysis

Eligible studies were analyzed using a random‐effects model to estimate weighted mean differences and 95% confidence intervals (CIs) between DAF and NAF. Studies missing key data (e.g., means, SDs, sample sizes) were excluded from the meta‐analysis. If multiple comparable DAF conditions (e.g., delay times of 50 ms, and 100 ms) were tested, each was coded separately. When estimating weighted mean difference and 95% CI at the study level, means and SD were combined into a single group in accordance with the Cochrane Handbook (Higgins et al. 2024) to avoid duplication. Statistical analyses and visualizations were conducted with Review Manager 5.4.1 (The Cochrane Collaboration 2020).

Heterogeneity was assessed using Cochrane's Q and I^2^ statistics (Higgins et al. 2003). Given that Cochrane's Q has limited sensitivity in small samples, I^2^ was also used to estimate the percentage of variance attributable to actual differences. Subgroup analyses compared outcomes by disfluency type (SLD vs. OD), delay time, speech task, stuttering severity, and participant age. Publication bias was examined using a funnel plot and statistical tests conducted in R 4.0.4 (R Core Team 2021).

## 3 Results

### 3.1 Article Search Process

**FIGURE 1:**
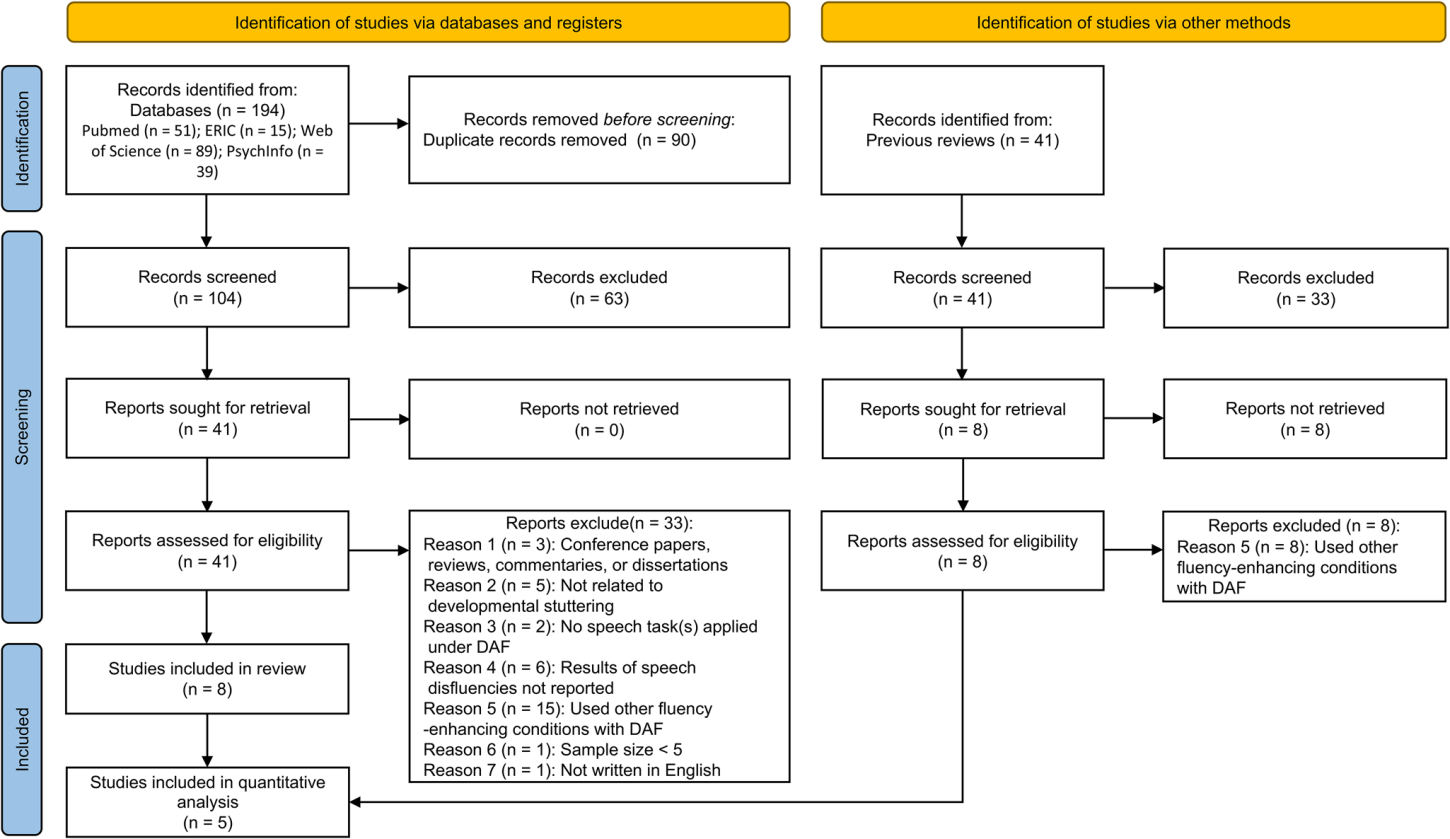
PRISMA 2020 flow diagram.

The article selection process is shown in Figure 1. A database search yielded 194 citations. After removing duplicates, 104 articles remained for Stage 1 screening. Of these, 63 were excluded based on eligibility criteria with κ = 0.66 (substantial interrater agreement; Landis and Koch 1977). Disagreements were resolved through discussion. In the full‐text screening, a total of 33 articles of 41 articles were excluded, resulting in a kappa of 0.50 (moderate agreement). Owing to insufficient reliability, a third author (DI) reviewed all remaining articles and resolved any disagreements through consensus discussion between all authors. A list of the excluded studies and the reasons for their exclusion is provided in the supplementary table. Although a manual literature search was conducted, none of the identified articles met the inclusion criteria. Therefore, a total of eight studies involving 98 participants were included.

### 3.2 Summary of the Included Articles

Participant information is summarized in Table 1. Sample sizes ranged from 8 to 20, with ages spanning school‐age children to adults in their 50s. Most participants were men, and most articles confirmed no presence of hearing, speech, or language problems. Some studies specified prior stuttering therapy, a diagnosis of developmental stuttering, or minimum severity thresholds (e.g., greater than 3%–5% SLD). Reported severity ranged from mild (Ishida et al. 2021) to severe (Van Borsel et al. 2003), with some samples spanning mild to severe cases (Fiorin et al. 2021; Stuart and Kalinowski 2004). It should be noted that these severity levels were assessed using different scales, that is, SSI, SSI‐3, Illinois Clinician Stuttering Severity Scale, or Scale for Rating severity of stuttering. Consequently, cross‐study comparisons of stuttering severity should be interpreted with caution because different severity scales were used.

**TABLE 1:** Summary of participants’ information in each study.

Authors (year)	Sample size	Age	Gender (%male)	Eligibility	Stuttering severity
Van Borsel et al. (2003)	9	Range:18–45 mean: 26;5	44.4	Reportedly right‐handed Normal hearing (better than 25 dB HL) Monolingual speakers of Dutch Had stuttering of developmental origin.	1 mild; 1 severe; 7 very severe (SSI)
Stuart and Kalinowski (2004)	10	Range:14–36 Mean: 21.1	80	NR	5 mild; 5 severe
Antipova et al. (2008)	8	Range:16–55 Mean: 35 SD:12.95	87.5	Not currently involved in any speech‐language therapy program Hearing sensitivity within normal limits (25 dB HL or better at frequencies from 250 to 8000 Hz) Not diagnosed with any speech or language disorders	1 very mild; 3 mild; 2 moderate; 2 severe (SSI‐3)
Saltuklaroglu et al. (2009)	10	Range:18–51 Mean: 30.42	90	Displayed at least 5% stuttered syllables in an uncontrolled reading task No diagnosis of any other speech, language, hearing, or motor disorder.	Not reported
Picoloto et al. (2017)	10^a^	Mean:11.80 SD:3.90	NR	Native speakers of Brazilian Portuguese Chronological age between seven and 17 years and 11 months Diagnosis of persistent developmental stuttering A minimum of 3% of SLDs Not the stuttering severity of very mild; a minimum score of 11 points (from seven to 16 years and 11 months) or 18 points (over 17 years) in the SSI‐3 Pure tone audiometry within normality patterns and tympanometric curve type A	Mean: 23.20, SD: 7.10 (Total Score of SSI in the NAF condition)
Chon et al. (2021)	15^b^	Range: 19–34 Mean: 23.4 SD: 4.2	73.3	Monolingual native English speakers Diagnosed as persistent stuttering Exhibited at least three SLDs per 100 syllables Demonstrated normal hearing (20db HR or better at frequencies of 500, 1000, 2000, and 4000 Hz) Right‐handed Had no known hearing, speech, language, or neurological disorders Naïve to DAF	7 mild; 7 moderate; 1 severe (Illinois Clinician Stuttering Severity Scale; Yairi and Ambrose 2005)
Fiorin et al. (2021)	16	Range: 8–17	68.8	The onset of stuttering occurred during childhood Duration of at least 36 months of SLD without remission Minimum 3% of SLDs Audiometric thresholds within the normal limits Tympanometry curve type A Present contralateral stapedial acoustic reflexes No history of conduction and/or neurological alterations Not currently attending speech‐language therapy sessions	8 moderate; 8 severe (SSI‐3)
Ishida et al. (2021)	20	Mean: 25.65 SD: 3.80	70	NR	13 very mild; 2 mild; 2 mild to moderate; 3 moderate (Scale for Rating severity of stuttering; Johnson et al. 1963)

Abbreviations: HL, hearing level; NAF, non‐altered feedback; NR, not reported; SD, standard deviation; SSI: SLD, stuttering‐like disfluency; SSI‐3, Stuttering Severity Instrument 3rd edition (Riley 1994); stuttering severity instrument (Riley 1972).

^a^ The 10 participants with stuttering and central auditory processing disorders were excluded from our study.

^b^ Report involved two studies, but Study2 was not included in our study because disfluencies were not analyzed.

Study characteristics are summarized in Table 2. Tasks included reading and spontaneous or monologue speech. DAF delays ranged from 50 ms (Antipova et al. 2008) to 250 ms (Chon et al. 2021). Several studies tested multiple delays (Antipova et al. 2008; Saltuklaroglu et al. 2009; Van Borsel et al. 2003) and combined DAF with other altered feedback conditions such as FAF (Antipova et al. 2008; Fiorin et al. 2021; Saltuklaroglu et al. 2009). Except for one longitudinal pre–post study (Van Borsel et al. 2003), all designs were cross‐sectional. Outcomes included SLD, OD, or total disfluencies.

**TABLE 2:** Summary of study overview in each study.

Authors (year)	Speech task	Delay time	Control	Independent variable(s)	Outcome(s)	Analysis
Van Borsel et al. (2003)	Automatic speech Reading aloud Repeating words and sentences Picture description Conversation	Pre: 93 Post:13(93)∼147^a^	NAF	NAF, DAF (pre‐exposure of DAF and post‐exposure of DAF during 3 months)	SLD (mean percentage of words stuttered)	Non‐parametric (Freedman's test, Wilcoxon signed‐rank test)
Stuart and Kalinowski (2004)	Read aloud	50	NAF	NAF, DAF	SLD (stuttering frequency)	No statistical analysis^b^
Antipova et al. (2008)	Monologue	50, 75 ^c^	NAF	NAF, DAF (50 ms), DAF (75 ms), DAF (50 ms) & FAF+1/5 octave, DAF (50 ms) & FAF‐1/3 octave, DAF (50 ms) & FAF‐1/2 octave, DAF (75 ms) & FAF+1/5 octave, DAF (75 ms) & FAF‐1/3 octave, DAF (75 ms) & FAF‐1/2 octave	SLD(%SS)	Parametric (*t*‐test or ANOVA)
Saltuklaroglu et al. (2009)	Read aloud	100, 200^c^	NAF	NAF, DAF (100 ms), DAF (200 ms), FAF+1/2 octave, FAF‐1/2 octave, choral	SLD(%SS)	Parametric (ANOVA)
Picoloto et al. (2017)	Spontaneous speech	100	NAF	NAF, DAF	SLD OD SLD+OD	Non‐parametric (Wilcoxon signed‐rank test and Mann‐Whitney test)
Chon et al. (2021)	Spontaneous speech	250	NAF	NAF, DAF	SLD OD	Parametric (*t*‐test)
Fiorin et al. (2021)	Spontaneous speech	100	NAF	NAF, DAF, MAF, AAF	SLD OD	Non‐parametric (Wilcoxon signed‐rank test and Mann–Whitney test)
Ishida et al. (2021)	Read aloud	200	NAF	NAF, DAF, NAF & auditory task, NAF & tactile task, DAF & auditory task, DAF & tactile task	SLD+OD	Parametric (ANOVA)

Abbreviations: %SS, proportion of stuttered syllables; AAF, amplified auditory feedback; ANOVA, analysis of variance; DAF, delayed auditory feedback; FAF, frequency altered feedback; MAF, masked auditory feedback; NAF, non‐altered feedback; OD, other disfluency; SLD, stuttering‐like disfluency.

^a^ Delay time differed across participants. The individual delay times were 147, 133, 13 (possibly a typographical error in the original study), 93, 93, 93, 93, 93, and 93 ms.

^b^ Given that speech disfluencies of participants were not a primary outcome of this study.

^c^ Implemented as separate conditions.

### 3.3 Main Comparisons Between DAF and NAF

Main comparisons between DAF and NAF are presented in Table 3. Five studies conducted statistical analyses to evaluate the effects of DAF relative to NAF. Results were inconsistent: three studies reported decreased disfluencies under DAF (Antipova et al. 2008; Saltuklaroglu et al. 2009; Stuart and Kalinowski 2004), whereas Ishida et al. (2021) reported increased disfluencies, and others reported mixed findings. Fiorin et al. (2021) observed reduced SLD only in participants with severe stuttering. Among studies focusing on disfluency type, one reported reduced SLD with DAF (Picoloto et al. 2017), one reported the reverse (Chon et al. 2021), and one found severity‐dependent effects (Fiorin et al. 2021). For OD, one study found a reduction (Fiorin et al. 2021), whereas two reported no effect (Chon et al. 2021; Picoloto et al. 2017). The only pre–post study (Van Borsel et al. 2003) reported disfluency reduction under DAF across tasks before therapy; however, the effects diminished post‐therapy, persisting only in the reading task.

**TABLE 3:** Main comparison of DAF and NAF conditions.

Authors (year)	Results of the degree of speech disfluencies in experimental conditions	Included in meta‐analysis	Extracted outcome for meta‐analysis
Van Borsel et al. (2003)	Pre‐exposure condition: DAF < NAF (*p* < 0.05) in all five tasks; Post‐exposure conditions: DAF < NAF (*p* = 0.011) in oral reading task; no significance in other four task	No	
Stuart and Kalinowski (2004)	No statistical analysis: For overall tendency, DAF 50 ms < NAF in both mild and severe stuttering groups	Yes	2 (stuttering severity: mild and severe)
Antipova et al. (2008)	Significant main effect of condition: DAF (75 ms) < NAF (*p* = 0.039) and DAF (50 ms) < NAF (*p* = 0.141)	No	
Saltuklaroglu et al. (2009)	No statistical analysis: For overall tendency, DAF (100 ms) < NAF and DAF 200 ms < NAF	Yes	2 (delay time: 100 and 200 ms)
Picoloto et al. (2017)	No statistical analysis; For overall tendency, DAF < NAF of SLD but reverse effect of OD. A noticeable difference has not been observed in the SLD+OD	Yes	2 (disfluency type: SLD and OD)
Chon et al. (2021)	Significancy in DAF > NAF of SLD (*p* < 0.001, Cohen's *d* = 1.205), while no difference of OD (*p* = 0.734)	Yes	2 (disfluency type: SLD and OD)
Fiorin et al. (2021)	Only severe stuttering group, DAF < NAF of SLD (*p* = 0.012); No significant difference in other combinations of stuttering severity group or disfluency type (SLD/OD) (*p* > 0.05)	Yes	2 (stuttering severity: mild and severe)^a^
Ishida et al. (2021)	Significancy in DAF > NAF of SLD+ND (*p* < 0.001, ${\eta_{G}}^{2}$= 0.439)	No	

Abbreviations: OD, other disfluency; SLD, stuttering‐like disfluency.

^a^ Only OD data were extracted because descriptive statistics for SLD were not reported.

### 3.4 Meta‐Analysis of the DAF Effect

**FIGURE 2:**
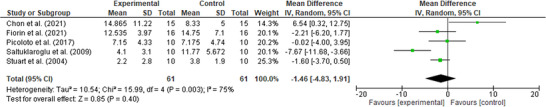
Forest plot of all outcomes.

Of the eight articles initially included, three (Antipova et al. 2008; Ishida et al. 2021; Van Borsel et al. 2003) were excluded because key descriptive statistics could not be extracted. Therefore, five studies involving a total of 61 participants contributed ten outcomes to the meta‐analysis. Three studies compared disfluency types (Chon et al. 2021; Fiorin et al. 2021; Picoloto et al. 2017), one examined delay times (Saltuklaroglu et al. 2009), and two assessed stuttering severity (Fiorin et al. 2021; Stuart and Kalinowski 2004). Studies with multiple groups were combined into single pairwise comparisons, and results were synthesized in a forest plot (Figure 2). The pooled mean difference between DAF and NAF was not significant (–1.46 [95% CI: –4.83, 1.91]). One study favored DAF (Saltuklaroglu et al. 2009), whereas Chon et al. (2021) favored NAF; indicating greater disfluency under DAF. The remaining studies reported nonsignificant results (Fiorin et al. 2021; Picoloto et al. 2017; Stuart and Kalinowski 2004). Heterogeneity was high (χ^2^ = 15.99, *p* = 0.003), warranting subgroup analyses. Publication bias (Appendix A) was assessed using statistical tests of rank correlation (Begg's test: *p* = 1.00) and regression (Egger's test: *p* = 0.281); however, the findings were inconclusive because of the small number of studies.

Subgroup results for disfluency type, delay time, speech task, stuttering severity, and participant age are presented in Appendices B–F. These analyses should be considered exploratory and hypothesis‐generating because several subgroups included only a small number of studies. For example, the reading subgroup included only two studies (Saltuklaroglu et al. 2009; Stuart and Kalinowski 2004), limiting the reliability of comparisons across speech tasks. The high heterogeneity observed across subgroups (χ^2^ = 6.47–31.67, *p* < 0.001–0.17) may reflect small sample sizes and potential confounding factors.

### 3.5 Quality Assessment

The quality assessment results are presented in Table 4. Most studies scored one point each for masking (described blind assessment or inter‐rater reliability), quantitative analysis, and confirmation of significance. None of the studies included a control group (score = 0). Only one study (Van Borsel et al. 2003) used a longitudinal design, earning a point in this category; thus, most of the included studies were cross‐sectional. One study (Stuart and Kalinowski 2004) received the lowest quality assessment score (1 point), corresponding to the 12.5th percentile of the quality scores reported by Andrade and Juste (2011). However, excluding this study did not alter the overall effect estimate (mean difference = –1.46, 95% CI [–4.83, 1.91]). Therefore, all eligible studies were retained in the meta‐analysis.

**TABLE 4:** Quality assessment of studies.

Authors (year)	Masking	Control group	Quantitative analysis	Confirmation of the significance	Longitudinal design	Total score
Van Borsel et al. (2003)	1	0	1	1	1	4
Stuart and Kalinowski (2004)	1	0	0	0	0	1
Antipova et al. (2008)	1	0	1	1	0	3
Saltuklaroglu et al. (2009)	1	0	1	1	0	3
Picoloto et al. (2017)	0	0	1	1	0	2
Chon et al. (2021)	1	0	1	1	0	3
Fiorin et al. (2021)	0	0	1	1	0	2
Ishida et al. (2021)	0	0	1	1	0	2
Percentage of scored ‘1’	62.5%	0%	87.5%	87.5%	12.5%	
Percentage of scored ‘1’ in previous review (Andrade and Juste 2011)	37.5%	12.5%	95.8%	79.2%	28.8%	

## 4 Discussion

### 4.1 Overall DAF Effect

This meta‐analysis synthesized evidence comparing disfluencies under DAF and NAF conditions, focusing on the exclusive effect of DAF. Findings showed no significant overall DAF effect. While numerous studies have examined the effects of altered auditory feedback on speech disfluencies in people who stutter (Andrade and Juste 2011; Lincoln et al. 2006), few investigated DAF alone. In fact, during full‐text screening, approximately half of the excluded studies (15/33) were eliminated because DAF was combined with other fluency‐enhancing conditions. Previous systematic reviews reported the utility of DAF in intervention studies (Baxter et al. 2015; Johnson et al. 2023), but most combined DAF with FAF (Andrade and Juste 2011; Antipova et al. 2008; Hudock and Kalinowski 2014; Lincoln et al. 2006; Lincoln et al. 2010; Ratyńska et al. 2012; Ritto et al. 2016; Stuart et al. 2004; Stuart and Kalinowski 2004; Unger et al. 2012). The present findings suggest that DAF alone may have limited effectiveness, consistent with recent research reporting no significant benefit (Alqhazo and Alkhamaiseh 2025). However, this does not preclude a potential DAF effect, as variability may exist across studies, as suggested by our subgroup analyses. These results underscore the need for further research to clarify DAF mechanisms and control for confounding factors.

### 4.2 Factors Related to the DAF Effect

Subgroup analyses revealed high heterogeneity and small sample sizes; thus, results must be interpreted with caution. Three factors repeatedly highlighted in prior research—delay time, stuttering severity, and speech task—are discussed below.

#### 4.2.1 Delay Time

In the present meta‐analysis, delay times ranged from 50 ms (Antipova et al. 2008) to 250 ms (Chon et al. 2021). Some studies have suggested that shorter delays (<100 ms) may reduce disfluencies, whereas longer delays (>200 ms) have been associated with mixed or sometimes adverse effects (Chon et al. 2021; Ishida et al. 2021). However, these observations are based on a small number of studies with differing methodologies and should therefore be interpreted cautiously. Early studies often employed longer initial delays (200–250 ms; Goldiamond 1965; Lincoln et al. 2006; Ryan and Van Kirk 1974), which were gradually reduced as fluency was maintained (Goldiamond 1965). Recent research frequently applies shorter delays (50–100 ms;; Alqhazo and Alkhamaiseh 2025; Antipova et al. 2008; Kalinowski et al. 1993; Lincoln et al. 2006; Stuart and Kalinowski 2004; Stuart et al. 2003). Kalinowski et al. (1996) suggested a minimum delay of 50 ms for maximum fluency benefit. Longer delays may induce speech rate reduction or prolonged speech, but this is not always necessary (Kalinowski et al. 1993, 1996; Stuart et al. 2003). In contrast, shorter delays appear to stabilize speech motor patterns (Chon et al. 2021). Increased linguistic complexity, such as longer utterances or greater phonological and syntactic demands, also elevates kinematic variability (Kleinow and Smith 2000; MacPherson and Smith 2013; Smith et al. 2010; Usler and Walsh 2018), which can trigger stuttering (Alqhazo and Al‐Dennawi 2018; Buhr and Zebrowski 2009; Coalson et al. 2012). From a motor control perspective (Civier et al. 2010; Max et al. 2004), shorter delays may better stabilize motor patterns and support fluency.

#### 4.2.2 Stuttering Severity

The present meta‐analysis did not find a significant difference in the effect of DAF according to stuttering severity. However, this finding should be interpreted cautiously based on the limited number of studies available for analysis. Several prior studies suggest greater benefits for individuals with severe stuttering (Foundas et al. 2013; Sparks et al. 2002; Unger et al. 2012). For example, Sparks et al. (2002) reported DAF effects only in severe cases, and Ishida et al. (2021) observed adverse effects in mild cases. In Fiorin et al. (2021), the data included in the meta‐analysis were limited to OD because insufficient descriptive statistics for SLD; nevertheless, they reported significant reductions in SLD among individuals with severe stuttering. In studies combining DAF and FAF, greater benefits were again observed in severe cases (Foundas et al. 2013; Unger et al. 2012). However, there are concerns regarding few benefits for individuals with mild stuttering. Previous studies suggested that mild stuttering may be subject to a floor effect (Antipova et al. 2008; Unger et al. 2012), where already low baseline disfluency leaves little room for further reduction. Overall, further research with larger samples is required to determine whether stuttering severity moderates the effects of DAF.

#### 4.2.3 Speech Task

Most studies used oral reading or spontaneous speech/monologue tasks, with only one including conversation (Van Borsel et al. 2003). Reading tasks were common in earlier research (Howell et al. 1999; Kalinowski et al. 1993, 1996; Lincoln et al. 2006; Stuart et al. 1997), whereas recent studies often use spontaneous speech or monologue (Antipova et al. 2008; Chon et al. 2021; Fiorin et al. 2021; Hudock and Kalinowski 2014; Lincoln et al. 2006; Picoloto et al. 2017; Ratyńska et al. 2012; Ritto et al. 2016; Stuart et al. 2004). In the present meta‐analysis, the reading‐task subgroup appeared to exhibit a larger DAF effect than the spontaneous speech subgroup, although this observation should be interpreted with caution because of the limited number of included studies and small sample sizes. Consistent with this tentative pattern, previous studies have reported that altered auditory feedback effects tend to be weaker for spontaneous speech than for reading tasks (Foundas et al. 2013; Lincoln et al. 2006). Studies using reading tasks consistently report reductions, whereas spontaneous speech tends to show limited effects (Armson and Stuart 1998; Armson et al. 2006; Foundas et al. 2013). While spontaneous speech involves greater linguistic complexity, including word selection and sentence formulation, Foundas et al. (2013) suggested that reading tasks may allow individuals to allocate more cognitive resources to feedback monitoring, thereby enhancing fluency. Although studies published before 2000 were not included in the present review, reading tasks were frequently used in earlier research. Reviewing and synthesizing findings from these earlier studies may help clarify whether task‐related differences in DAF effects are robust.

### 4.3 Limitations and Future Implications

This meta‐analysis focused on studies published since 2000. Given the small number of eligible studies and the frequent use of combined DAF and FAF in clinical contexts, earlier studies may still inform interpretation, though their contribution could remain limited due to methodological variability (Andrade and Juste 2011). To better clarify DAF mechanisms and obtain more reliable effect estimates, future studies should isolate DAF, incorporate subgroup analyses, and account for possible stuttering subtypes such as auditory processing disorders (Picoloto et al. 2017) and neuroanatomical abnormalities. In addition, the generalizability of the findings may be limited because this review relied exclusively on English‐language literature; consequently, relevant studies published in other languages may not have been captured. Furthermore, the review protocol was not registered in advance. Future updates of this meta‐analysis should include prospective protocol registration to enhance transparency and methodological rigor.

Several statistical and methodological limitations should be considered when interpreting the present findings. First, the number of studies included in the meta‐analysis was small, which restricts the reliability of several supplementary analyses. The subgroup analyses should therefore be interpreted as exploratory, because several subgroups consisted of only two studies. Second, the assessment of publication bias should also be interpreted cautiously. Although Begg's and Egger's tests did not indicate clear publication bias, these tests have very low statistical power when only a small number of studies are available. Overall, few number of eligible studies could limit the interpretation of the present meta‐analysis and highlights the need for additional research on the effects of DAF. Third, risk of bias was not formally assessed using established tools such as the Risk of Bias in Non‐randomised Studies of Interventions (ROBINS‐I; Sterne et al. 2016). Instead, study quality was evaluated using criteria proposed in a previous systematic review of stuttering and DAF (Andrade and Juste 2011). Some items in this framework had limited discriminative value. For example, all included studies received a score of 0 on the control‐group criterion (Table 4). Although the inclusion of a control group is important in intervention studies because it helps reduce the influence of confounding factors unrelated to the intervention itself, most intervention studies in this area involved SpeechEasy, which combines DAF and FAF and was therefore excluded from the present review. Consequently, the control‐group criterion contributed little to the assessment of study quality among the studies included in this review.

Future research should also emphasize methodological consistency, including control groups, blinding, task replication, and device calibration (Andrade and Juste 2011). Lincoln et al. (2006) highlighted the importance of conversational speech sampling to generalize findings beyond clinical and laboratory contexts. Yet, none of the studies in our meta‐analysis investigated conversational tasks after Lincoln's publication, despite later DAF+FAF studies (e.g., SpeechEasy) including such tasks (Armson and Stuart 1998; Armson et al. 2006; Foundas et al. 2013; Hudock and Kalinowski 2014; Lincoln et al. 2010; O'Donnell et al. 2008; Pollard et al. 2009; Ritto et al. 2016). Although spontaneous speech is challenging to control due to confounding variables, further research on DAF in conversational contexts remains essential.

Interactions between factors also merit attention. For instance, optimal delay time may depend on the speech task. Antipova et al. (2008) found that longer delays produced greater benefits for spontaneous speech or monologue than for reading. During spontaneous speech, controlling speech rate appears essential; the stronger the DAF‐induced slowing effect, the greater the potential improvement in fluency. Further validation of these interactions would be instrumental in taking advantage of the individualize DAF effect for stuttering.

## 5 Conclusion

This meta‐analysis is the first to systematically evaluate the exclusive effect of DAF on stuttering. The results indicate that DAF alone does not significantly reduce disfluency. The included studies involved sample sizes ranging from 8 to 20 participants, with ages spanning from school‐age children to adults. Most participants were male, and stuttering severity ranged from mild to severe. DAF was evaluated using oral reading and spontaneous speech/monologue tasks. The findings also highlight the heterogeneous nature of DAF effects and underscore the need for studies with sufficient sample size and better‐controlled design to clarify its therapeutic potential.

## Funding

This work was supported by the University of Tsukuba Basic Research Support Program Type S.

## Conflicts of Interest

The authors declare no conflicts of interest.

## Supporting information




**Supporting file**: jlcd70283‐supp‐0001‐SuppMat.docx

**Supporting file**: jlcd70283‐supp‐0002‐SuppMat.TIF

**Supporting file**: jlcd70283‐supp‐0003‐SuppMat.TIF

**Supporting file**: jlcd70283‐supp‐0004‐SuppMat.TIF

**Supporting file**: jlcd70283‐supp‐0005‐SuppMat.TIF

**Supporting file**: jlcd70283‐supp‐0006‐SuppMat.TIF

**Supporting file**: jlcd70283‐supp‐0007‐SuppMat.TIF

## Data Availability

The data that support the findings of this study are available from the corresponding author upon reasonable request.
