# Supplementary material for: Immediate Effects of Delayed Auditory Feedback on Stuttering: A Systematic Review and Meta‐Analysis of Literature Published 2000–2024
Source: Int J Lang Commun Disord. 2026 Jun 24;61(4):e70283. doi: 10.1111/1460-6984.70283 (PMC13292190; doi:10.1111/1460-6984.70283)
Supplement: Supplementary file 1 — Supporting file: jlcd70283‐supp‐0001‐SuppMat.docx [file JLCD-61-0-s001.docx]

Supplementary Table: Excluded studies and reasons for exclusion

| No. | Article | Reasons for exclusion |
| --- | --- | --- |
| 1 | Alm, P. A., & Risberg, J. (2007). Stuttering in adults: The acoustic startle response, temperamental traits, and biological factors. Journal of Communication Disorders, 40(1), 1–41. | Reason 4: Results of speech disfluencies not reported |
| 2 | Bowyer, S. M., Peacock, J., Tepley, N., & Moran, J. E. (2010). Neuronal effects of the SpeechEasy treatment for stuttering. In 17th International Conference on Biomagnetism Advances in Biomagnetism, pp. 342–345, Croatia . | Reason 1: Conference papers, reviews, commentaries, or dissertations |
| 3 | Daliri, A., & Max, L. (2018). Stuttering adults' lack of pre-speech auditory modulation normalizes when speaking with delayed auditory feedback. Cortex, 99, 55–68. | Reason 4: Results of speech disfluencies not reported |
| 4 | Dayalu, V. N., Kalinowski, J., & Saltuklaroglu, T. (2002). Active inhibition of stuttering results in pseudofluency: A reply to Craig. Perceptual and Motor Akills, 94(3), 1050–1052. | Reason 1: Conference papers, reviews, commentaries, or dissertations |
| 5 | Foundas, A. L., Bollich, A. M., Feldman, J., Corey, D. M., Hurley, M., Lemen, L. C., & Heilman, K. M. (2004). Aberrant auditory processing and atypical planum temporale in developmental stuttering. Neurology, 63(9), 1640–1646. | Reason 5: Used other fluency-enhancing conditions with DAF |
| 6 | Hudock, D., Dayalu, V. N., Saltuklaroglu, T., Stuart, A., Zhang, J., & Kalinowski, J. (2015). Stuttering inhibition via visual feedback at normal and fast speech rates. International Journal of Language & Communication Disorders, 1–10. | Reason 3: No speech task(s) applied under DAF |
| 7 | Hudock, D., & Kalinowski, J. (2014). Stuttering inhibition via altered auditory feedback during scripted telephone conversations. International Journal of Language & Communication Disorders, 49(1), 139–147. | Reason 5: Used other fluency-enhancing conditions with DAF |
| 8 | Iimura, D., Asakura, N., Sasaoka, T., & Inui, T. (2019). Abnormal sensorimotor integration in adults who stutter: A behavioral study by adaptation of delayed auditory feedback. Frontiers in Psychology, 10, 2440. | Reason 4: Results of speech disfluencies not reported |
| 9 | Kalinowski, J. (2003). Self-reported efficacy of an all in-the-ear-canal prosthetic device to inhibit stuttering during one hundred hours of university teaching: An autobiographical clinical commentary. Disability and Rehabilitation, 25(2), 107–111. | Reason 3: No speech task(s) applied under DAF |
| 10 | Lazzari, G., van de Vorst, R., van Vugt, F. T., & Lega, C. (2024). Subtle Patterns of Altered Responsiveness to Delayed Auditory Feedback during Finger Tapping in People Who Stutter. Brain Sciences, 14(5), 472. | Reason 5: Used other fluency-enhancing conditions with DAF |
| 11 | Lincoln, M., & Walker, C. (2007). A survey of Australian adult users of altered auditory feedback devices for stuttering: use patterns, perceived effectiveness and satisfaction. Disability and Rehabilitation, 29(19), 1510–1517. | Reason 5: Used other fluency-enhancing conditions with DAF |
| 12 | Lincoln, M., Packman, A., Onslow, M., & Jones, M. (2010). An experimental investigation of the effect of altered auditory feedback on the conversational speech of adults who stutter. Journal of Speech, Language, and Hearing Research, 53(5), 1122–1131. | Reason 5: Used other fluency-enhancing conditions with DAF |
| 13 | Moein, N., Mohamadi, R., Rostami, R., Nitsche, M., Zomorrodi, R., & Ostadi, A. (2022). Investigation of the effect of delayed auditory feedback and transcranial direct current stimulation (DAF-tDCS) treatment for the enhancement of speech fluency in adults who stutter: A randomized controlled trial. Journal of Fluency Disorders, 72, 105907. | Reason 5: Used other fluency-enhancing conditions with DAF |
| 14 | Moein, N., Mohamadi, R., Rostami, R., Nitsche, M., Zomorrodi, R., Ostadi, A., & Keshtkar, A. (2020). Delayed auditory feedback and transcranial direct current stimulation treatment for the enhancement of speech fluency in adults who stutter: protocol for a randomized controlled trial. JMIR Research Protocols, 9(4), e16646. | Reason 4: Results of speech disfluencies not reported |
| 15 | Natke U. (2000). Stotterreduktion bei frequenzverschobener und verzögerter auditiver Rückmeldung. Folia Phoniatrica et Logopaedica, 52 (4), 151–159. | Reason 7: Not written in English |
| 16 | Ozker, M., Doyle, W., Devinsky, O., & Flinker, A. (2022). A cortical network processes auditory error signals during human speech production to maintain fluency. PLoS Biology, 20(2), e3001493. | Reason 2: Not related to developmental stuttering |
| 17 | Raczek, B., & Adamczyk, B. (2004). Concentration of carbon dioxide in exhaled air in fluent and non-fluent speech. Folia Phoniatrica et Logopaedica, 56(2), 75–82. | Reason 5: Used other fluency-enhancing conditions with DAF |
| 18 | Radford, N. T., Tanguma, J., Gonzalez, M., Nericcio, M. A., & Newman, D. G. (2005). A case study of mediated learning, delayed auditory feedback, and motor repatterning to reduce stuttering. Perceptual and Motor Skills, 101(1), 63–71. | Reason 5: Used other fluency-enhancing conditions with DAF |
| 19 | Ratyńska, J., Szkiełkowska, A., Markowska, R., Kurkowski, M., Mularzuk, M., & Skarżyński, H. (2012). Immediate speech fluency improvement after application of the Digital Speech Aid in stuttering patients. Medical Science Monitor: International Medical Journal of Experimental and Clinical Research, 18(1), CR9. | Reason 5: Used other fluency-enhancing conditions with DAF |
| 20 | Ritto, A. P., Juste, F. S., Stuart, A., Kalinowski, J., & de Andrade, C. R. F. (2016). Randomized clinical trial: the use of SpeechEasy® in stuttering treatment. International Journal of Language & Communication Disorders, 51(6), 769–774. | Reason 5: Used other fluency-enhancing conditions with DAF |
| 21 | Sakai, N., Masuda, S., Shimotomai, T., & Mori, K. (2009). Brain activation in adults who stutter under delayed auditory feedback: An fMRI study. International Journal of Speech-Language Pathology, 11(1), 2–11. | Reason 5: Used other fluency-enhancing conditions with DAF |
| 22 | Saldanha, J. C., & Pinto, R. (2023). Real time feedback system for speech dysfluency in children. In Smart Sensors Measurement and Instrumentation: Select Proceedings of CISCON 2021, pp. 75–91. Singapore. | Reason 1: Conference papers, reviews, commentaries, or dissertations |
| 23 | Sparks, G., Grant, D. E., Millay, K., Walker-Batson, D., & Hynan, L. S. (2002). The effect of fast speech rate on stuttering frequency during delayed auditory feedback. Journal of Fluency Disorders, 27(3), 187–201. | Reason 6: Sample size < 5 |
| 24 | Stidham, K. R., Olson, L., Hillbratt, M., & Sinopoli, T. (2006). A new antistuttering device: treatment of stuttering using bone conduction stimulation with delayed temporal feedback. The Laryngoscope, 116(11), 1951–1955. | Reason 5: Used other fluency-enhancing conditions with DAF |
| 25 | Stuart, A., Kalinowski, J., Rastatter, M. P., Saltuklaroglu, T., & Dayalu, V. (2004). Investigations of the impact of altered auditory feedback in‐the‐ear devices on the speech of people who stutter: initial fitting and 4‐month follow‐up. International Journal of Language & Communication Disorders, 39(1), 93–113. | Reason 5: Used other fluency-enhancing conditions with DAF |
| 26 | Takaso, H., Eisner, F., Wise, R. J., & Scott, S. K. (2010). The effect of delayed auditory feedback on activity in the temporal lobe while speaking: a positron emission tomography study. Journal of Speech, Language, and Hearing Research, 53(2), 226–236. | Reason 2: Not related to developmental stuttering |
| 27 | Toyomura, A., & Omori, T. (2005). Auditory feedback control during a sentence-reading task: Effect of other’s voice. Acoustical Science and Technology, 26(4), 358–361. | Reason 2: Not related to developmental stuttering |
| 28 | Toyomura, A., Miyashiro, D., Kuriki, S., & Sowman, P. F. (2020). Speech-induced suppression for delayed auditory feedback in adults who do and do not stutter. Frontiers in Human Neuroscience, 14, 150. | Reason 4: Results of speech disfluencies not reported |
| 29 | Unger, J. P., Glück, C. W., & Cholewa, J. (2012). Immediate effects of AAF devices on the characteristics of stuttering: A clinical analysis. Journal of Fluency Disorders, 37(2), 122–134. | Reason 5: Used other fluency-enhancing conditions with DAF |
| 30 | Van Borsel, J., & Eeckhout, H. (2008). The speech naturalness of people who stutter speaking under delayed auditory feedback as perceived by different groups of listeners. Journal of Fluency Disorders, 33(3), 241–251. | Reason 4: Results of speech disfluencies not reported |
| 31 | Van Borsel, J., Sunaert, R., & Engelen, S. (2005). Speech disruption under delayed auditory feedback in multilingual speakers. Journal of Fluency Disorders, 30(3), 201–217. | Reason 2: Not related to developmental stuttering |
| 32 | Yamamoto, K., & Kawabata, H. (2011). Temporal recalibration in vocalization induced by adaptation of delayed auditory feedback. PLoS One, 6(12), e29414. | Reason 2: Not related to developmental stuttering |
| 33 | Yoshikawa, Y., Kobayashi, H., Sakai, N., Ishiguro, H., & Kumazaki, H. (2024). Therapeutic potential of robots for people who stutter: a preliminary study. Frontiers in Psychiatry, 15, 1298626. | Reason 5: Used other fluency-enhancing conditions with DAF |
| 34 | Armson, J., Kiefte, M., Mason, J., & De Croos, D. (2006). The effect of SpeechEasy on stuttering frequency in laboratory conditions. Journal of Fluency Disorders, 31(2), 137–152. | Reason 5: Used other fluency-enhancing conditions with DAF |
| 35 | Armson, J., & Kiefte, M. (2008). The effect of SpeechEasy on stuttering frequency, speech rate, and speech naturalness. Journal of Fluency Disorders, 33(2), 120–134. | Reason 5: Used other fluency-enhancing conditions with DAF |
| 36 | O’Donnell, J. J., Armson, J., & Kiefte, M. (2008). The effectiveness of SpeechEasy during situations of daily living. Journal of Fluency Disorders, 33(2), 99–119. | Reason 5: Used other fluency-enhancing conditions with DAF |
| 37 | Kalinowski, J., Guntupalli, V. K., Stuart, A., & Saltuklaroglu, T. (2004). Self-reported efficacy of an ear-level prosthetic device that delivers altered auditory feedback for the management of stuttering. International Journal of Rehabilitation Research, 27(2), 167–170. | Reason 5: Used other fluency-enhancing conditions with DAF |
| 38 | Kiefte, M., & Armson, J. (2008). Dissecting choral speech: Properties of the accompanist critical to stuttering reduction. Journal of Communication Disorders, 41(1), 33–48. | Reason 5: Used other fluency-enhancing conditions with DAF |
| 39 | Pollard, R., Ellis, J. B., Finan, D., & Ramig, P. R. (2009). Effects of the SpeechEasy on objective and perceived aspects of stuttering: a 6-month, phase I clinical trial in naturalistic environments. Journal of Speech, Language, and Hearing Research, 52(2), 516–533. | Reason 5: Used other fluency-enhancing conditions with DAF |
| 40 | Stuart, A., Kalinowski, J., Rastatter, M. P., Saltuklaroglu, T., & Dayalu, V. (2004). Investigations of the impact of altered auditory feedback in-the-ear devices on the speech of people who stutter: initial fitting and 4‐month follow‐up. International Journal of Language & Communication Disorders, 39(1), 93–113. | Reason 5: Used other fluency-enhancing conditions with DAF |
| 41 | Stuart, A., Kalinowski, J., Saltuklaroglu, T., & Guntupalli, V. K. (2006). Investigations of the impact of altered auditory feedback in-the-ear devices on the speech of people who stutter: One-year follow-up. Disability and Rehabilitation, 28(12), 757–765. | Reason 5: Used other fluency-enhancing conditions with DAF |
| Note. Articles No. 1–33 were identified through database searching, whereas Articles No. 34–41 were identified through manual searching. | | |
