# Supplementary figures and images for: Immediate Effects of Delayed Auditory Feedback on Stuttering: A Systematic Review and Meta‐Analysis of Literature Published 2000–2024
Source: Int J Lang Commun Disord. 2026 Jun 24;61(4):e70283. doi: 10.1111/1460-6984.70283 (PMC13292190; doi:10.1111/1460-6984.70283)

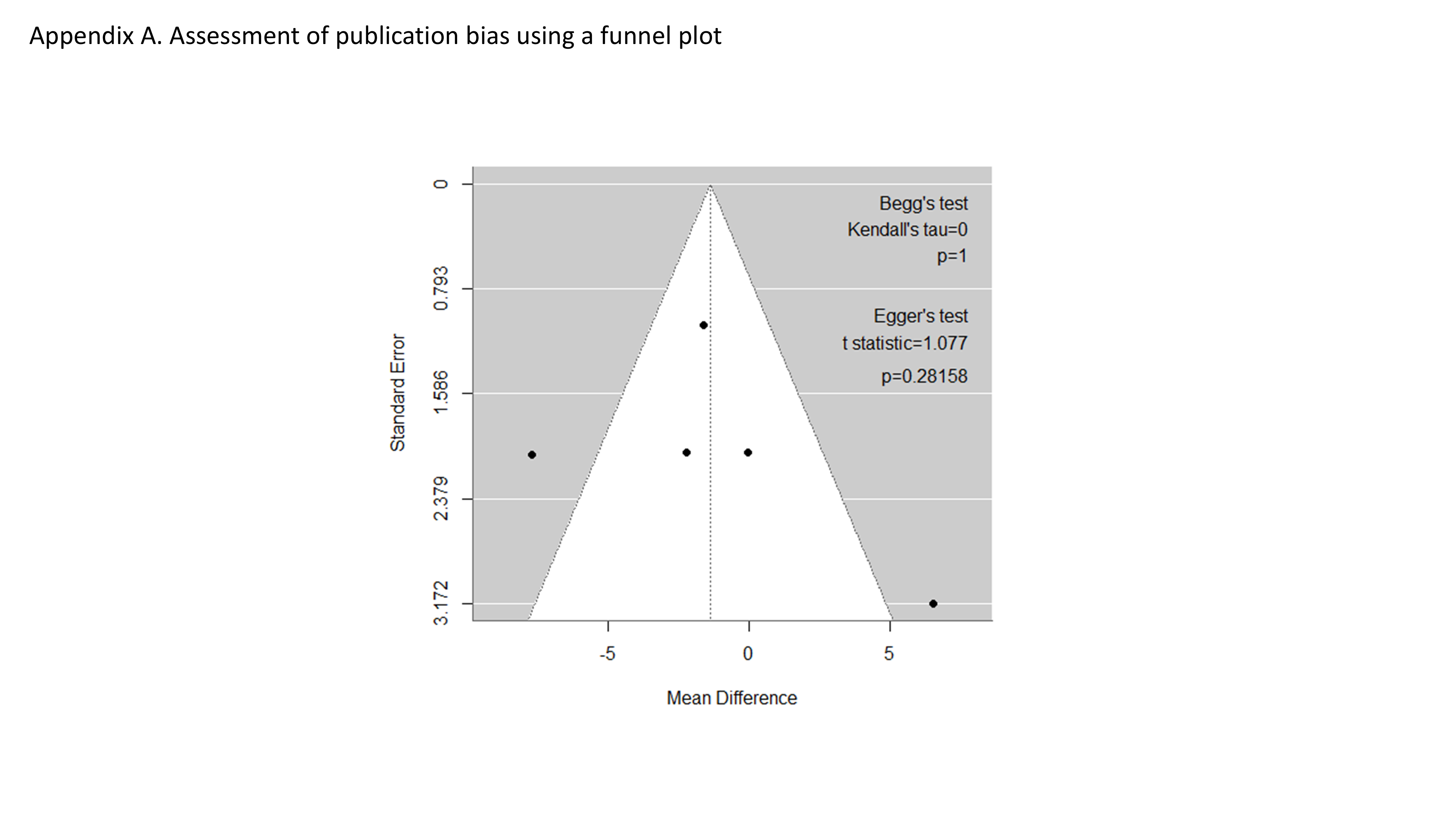

Supplement: Supplementary file 2 — Supporting file: jlcd70283‐supp‐0002‐SuppMat.TIF [file JLCD-61-0-s004.TIF]

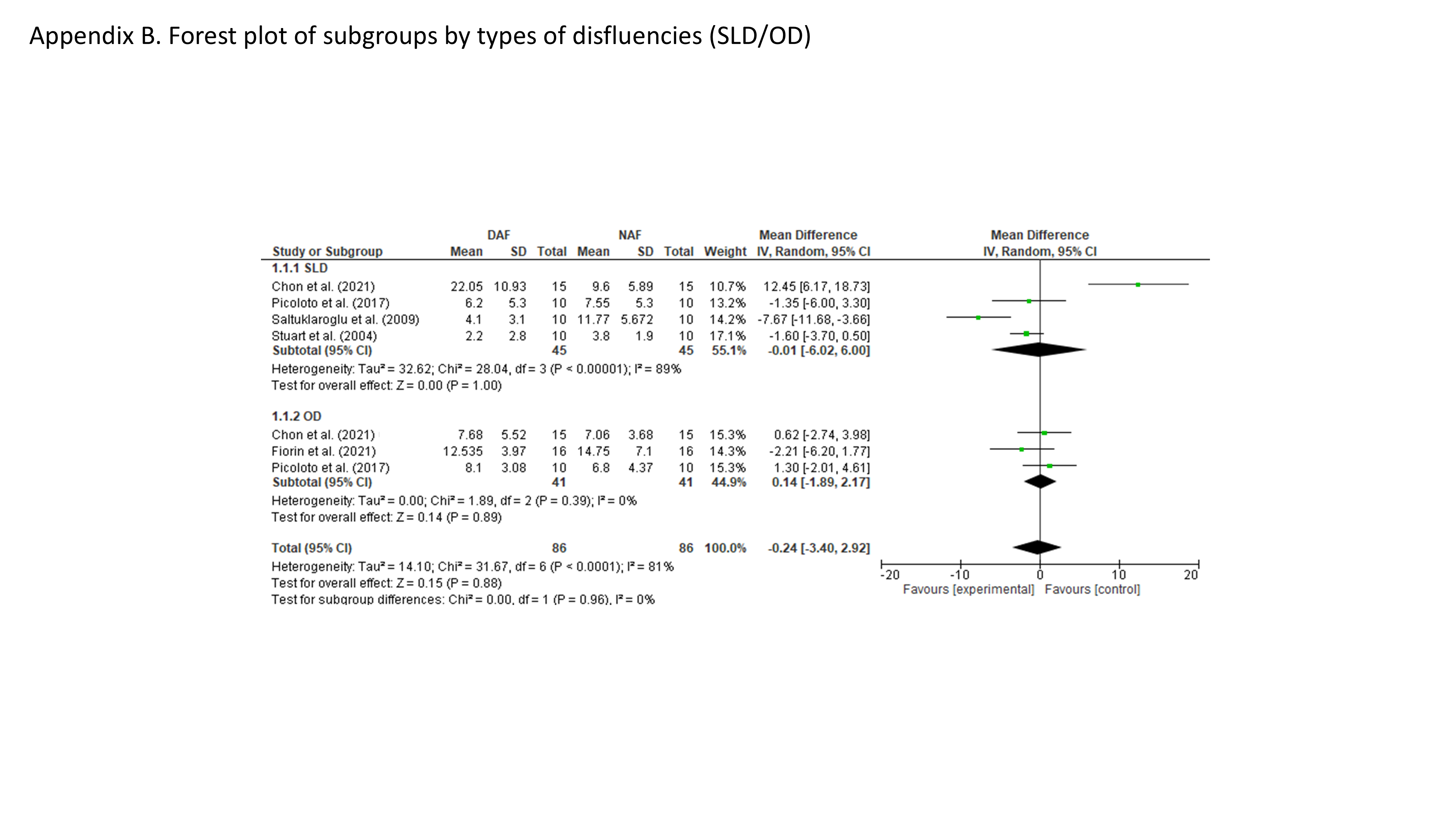

Supplement: Supplementary file 3 — Supporting file: jlcd70283‐supp‐0003‐SuppMat.TIF [file JLCD-61-0-s003.TIF]

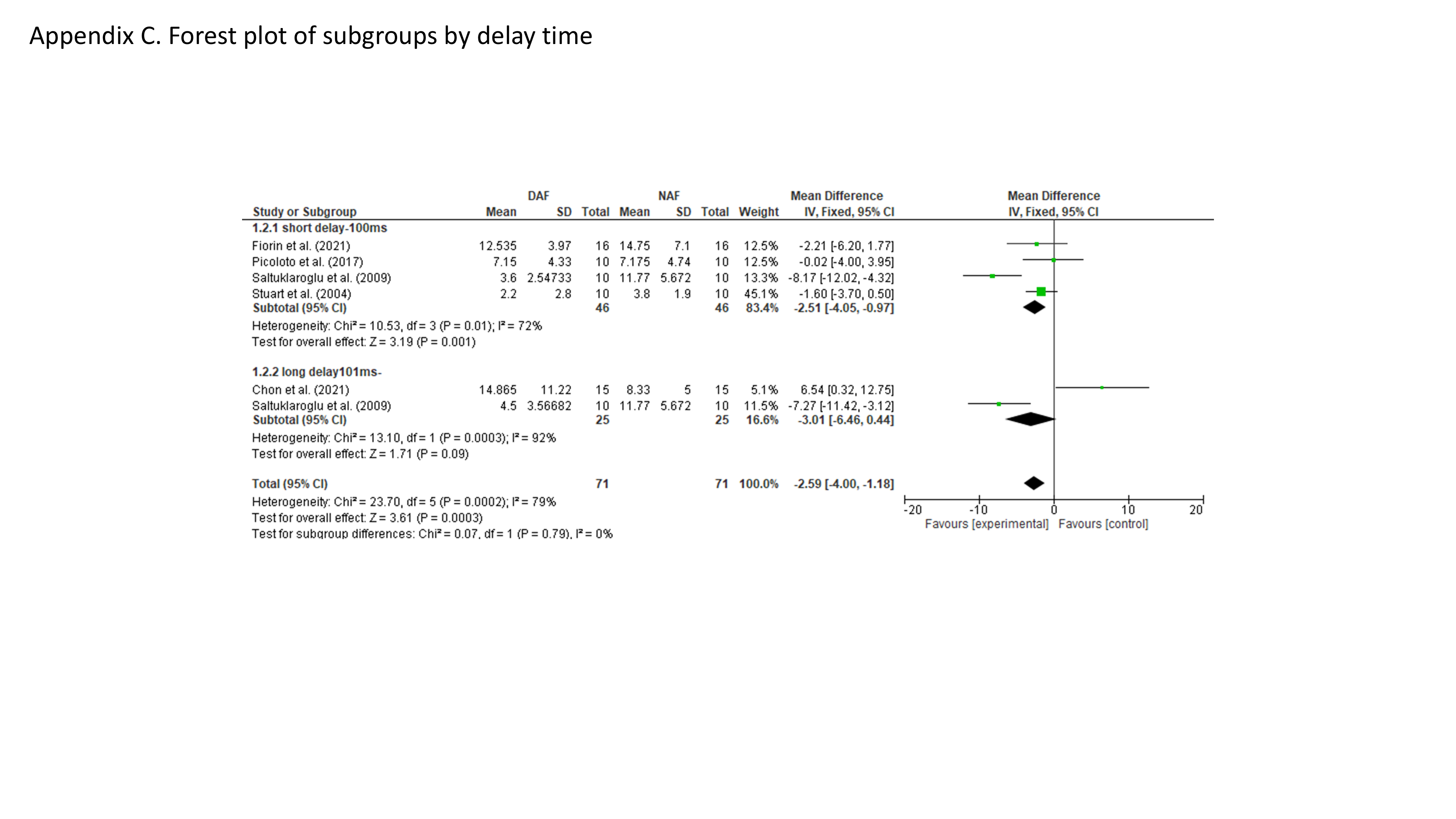

Supplement: Supplementary file 4 — Supporting file: jlcd70283‐supp‐0004‐SuppMat.TIF [file JLCD-61-0-s005.TIF]

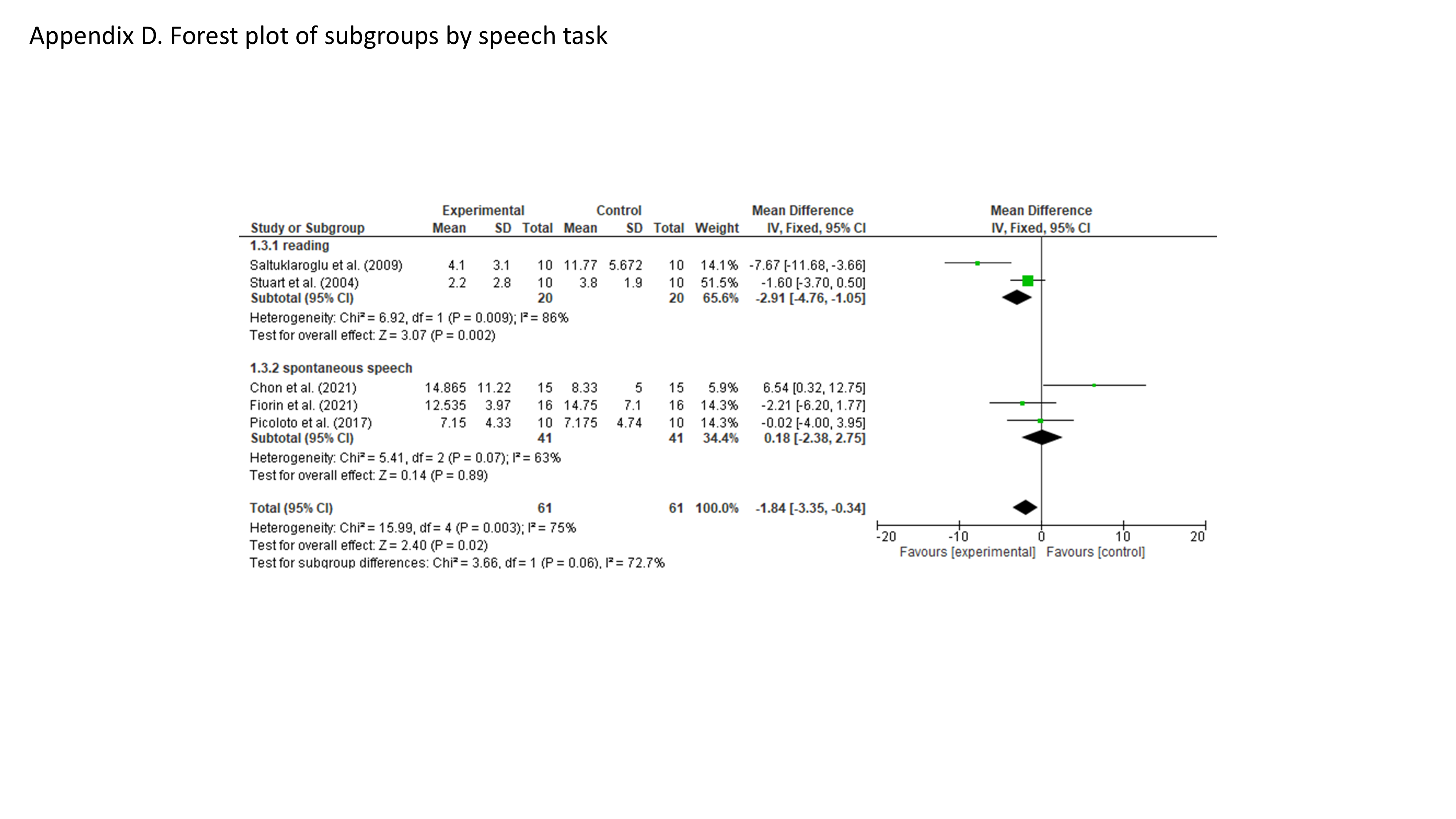

Supplement: Supplementary file 5 — Supporting file: jlcd70283‐supp‐0005‐SuppMat.TIF [file JLCD-61-0-s006.TIF]

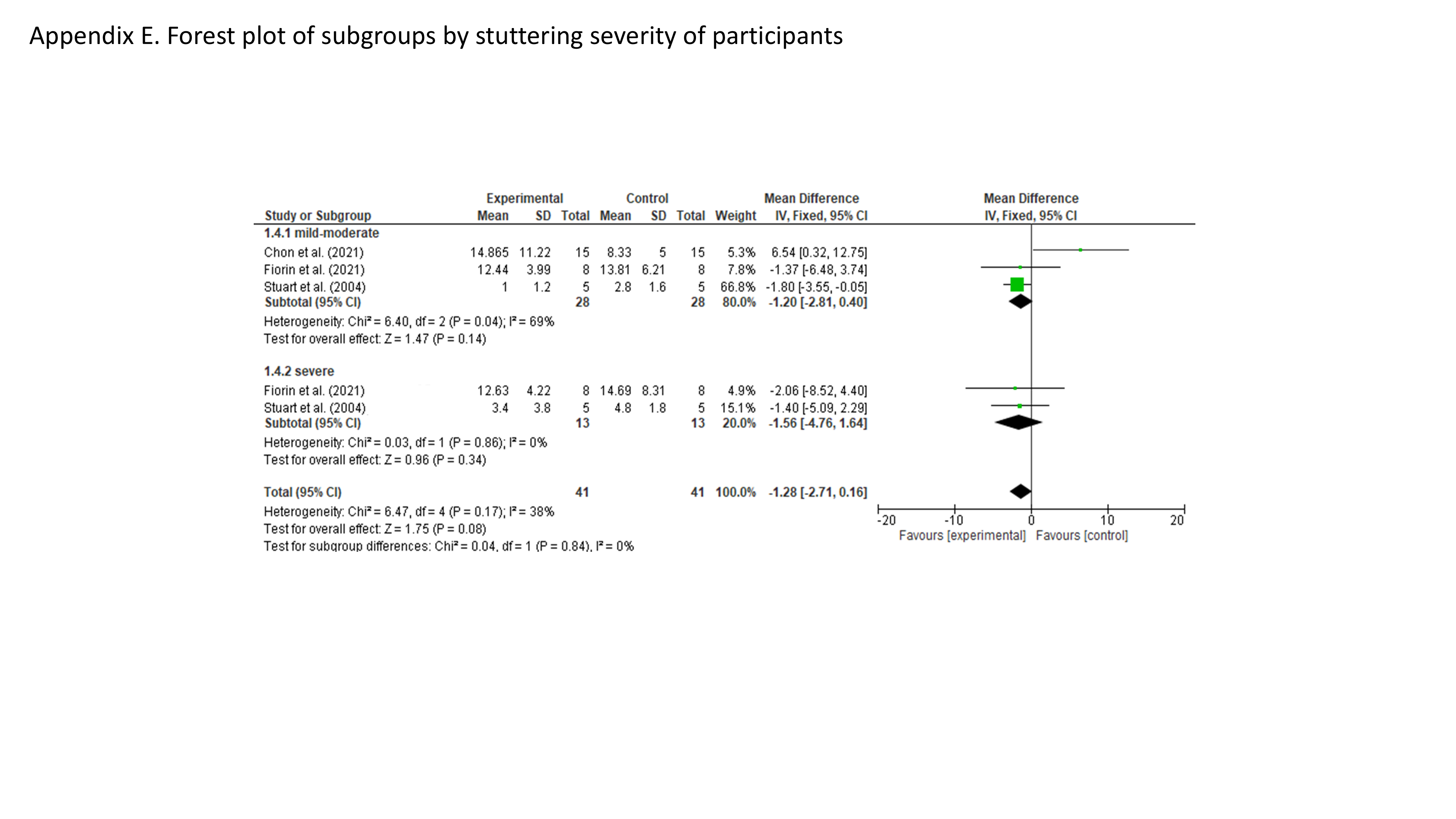

Supplement: Supplementary file 6 — Supporting file: jlcd70283‐supp‐0006‐SuppMat.TIF [file JLCD-61-0-s002.TIF]

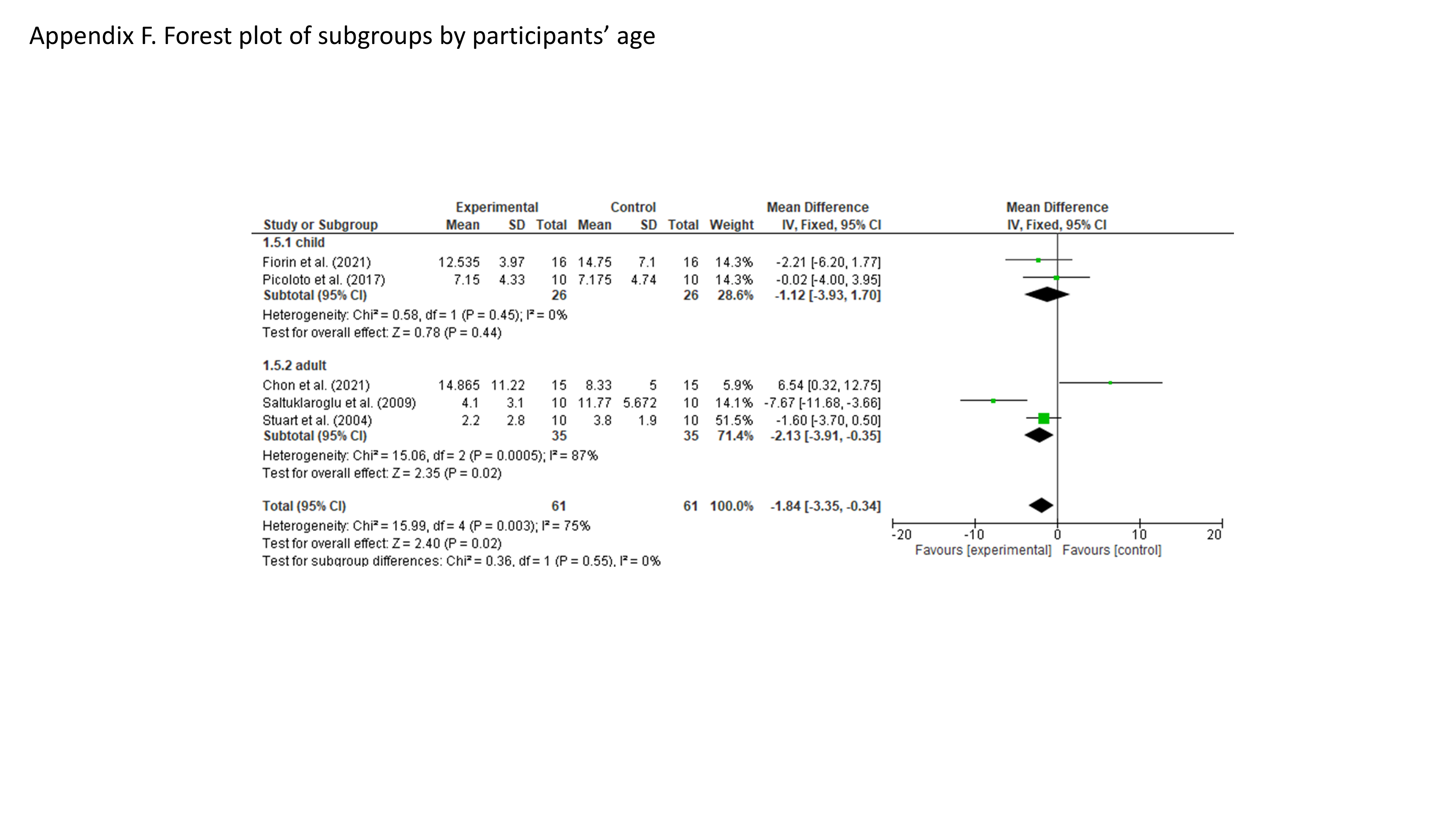

Supplement: Supplementary file 7 — Supporting file: jlcd70283‐supp‐0007‐SuppMat.TIF [file JLCD-61-0-s007.TIF]
